# Effective Elimination and Biodegradation of Polycyclic Aromatic Hydrocarbons from Seawater through the Formation of Magnetic Microfibres

**DOI:** 10.3390/ijms22010017

**Published:** 2020-12-22

**Authors:** M. Susana Gutiérrez, Alberto J. León, Paulino Duel, Rafael Bosch, M. Nieves Piña, Jeroni Morey

**Affiliations:** 1 Department of Chemistry, University of the Balearic Islands, Crta. de Valldemossa, Km. 7.5, 07122 Palma de Mallorca, Spain; dominika71@hotmail.com (M.S.G.); albertoperezleon@hotmail.com (A.J.L.); paulino.duel@uib.es (P.D.); jeroni.morey@uib.es (J.M.); 2 Department of Biology, University of the Balearic Islands, Crta. de Valldemossa, Km. 7.5, 07122 Palma de Mallorca, Spain; 3 Environmental Microbiology, IMEDEA (CSIC-UIB), Miquel Marquès, 21, 07190 Esporles, Spain

**Keywords:** magnetite nanoparticles, polycyclic aromatic hydrocarbon, microfibres, elimination, biodegradation

## Abstract

Supramolecular aggregates formed between polycyclic aromatic hydrocarbons and either naphthalene or perylene-derived diimides have been anchored in magnetite magnetic nanoparticles. The high affinity and stability of these aggregates allow them to capture and confine these extremely carcinogenic contaminants in a reduced space. In some cases, the high cohesion of these aggregates leads to the formation of magnetic microfibres of several microns in length, which can be isolated from the solution by the direct action of a magnet. Here we show a practical application of bioremediation aimed at the environmental decontamination of naphthalene, a very profuse contaminant, based on the uptake, sequestration, and acceleration of the biodegradation of the formed supramolecular aggregate, by the direct action of a bacterium of the lineage Roseobacter (biocompatible with nanostructured receptors and very widespread in marine environments) without providing more toxicity to the environment.

## 1. Introduction

**Figure 1:**
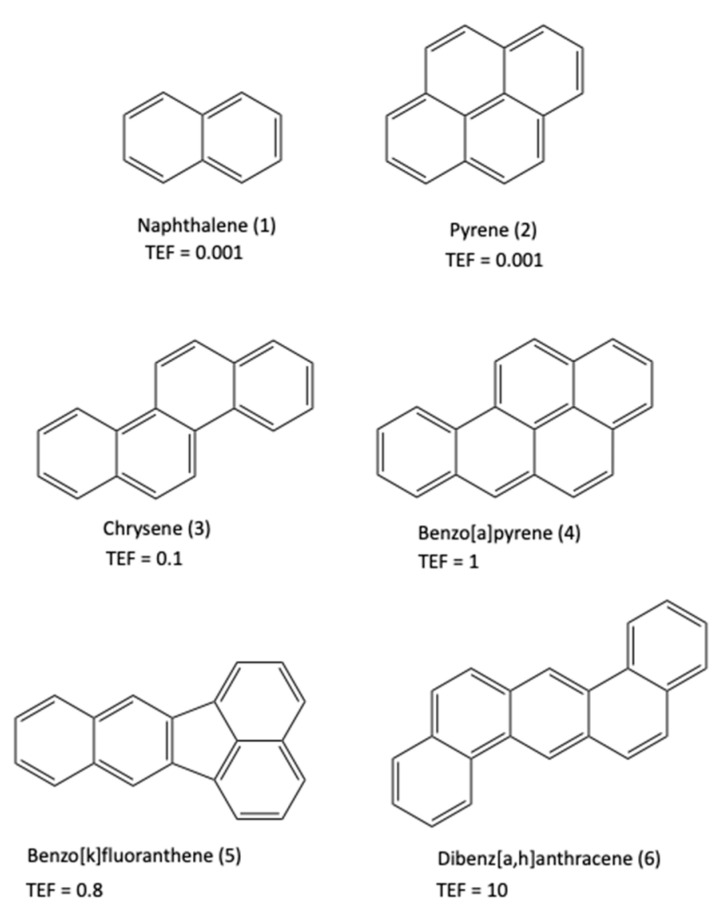
Polycyclic aromatic hydrocarbons used in this study.

The combined and synergistic use of nanotechnology, together with the action of microorganisms originating in the environment, can be a successful combination and a great help to accelerate and improve the overall process of natural bioremediation. The most worrying pollutants for the environment and its integral health have in common a high persistence and toxicity. Removing them completely and recycling and/or degrading them properly, safely, and effectively are not easy tasks. This work must be undertaken from the molecular perspective, developing molecular receptors capable of helping in this task without contributing to the toxicity of the environment. As a primary condition, these receptors must act in demanding environments and form robust supramolecular complexes. In this investigation, we describe the preparation of new hybrid nanoparticles formed by magnetite nanoparticles, Fe_3_O_4_, FeNP, linked the diimide-dopamine ligands prepared from four electron-deficient π-extended diimides, derived from benzene (BDI), naphthalene (NDI), perylene (PDI), and tetrabromodiimideperylene (BrPDI). This new hybrid nanomaterial is sensitive to the action of an external magnetic field, which makes possible its removal from a liquid suspension in a simple and comfortable way. From the point of view of reuse and sustainability with the environment, this magnetic property is very attractive, precisely because of its simplicity and effectiveness. Polycyclic aromatic hydrocarbons (PAHs) form a group of pollutants considered very dangerous for the environment. They are extremely carcinogenic and persistent contaminants [1,2,3,4,5]. In fact, since 1976, the U.S. Environmental Protection Agency (EPAH) advises a rigorous control of 16 specific PAHs, which can be generally detected in drinking water and also in certain foods that have a high percentage of oils and fats in their composition. The Toxicity Equivalence Factor (TEF, Figure 1) is used to compare the degree of toxicity among PAHs. For benzo[a]-pyrene (4) the TEF is 1, while the remaining PAHs in the list have values less than 1, except for dibenzo[a,h]-anthracene (6) with TEF = 10 [6]. Despite its low TEF (0.001), naphthalene is the most worrying of the PAHs because it is the most profuse and soluble anthropogenic PAH in water. Therefore, its control is a priority.

The members of the Roseobacter lineage (*Rhodobacteracea* family, *alphaproteobacteria* class) are predominant in the marine ecosystems, where they are ubiquitous, and they represent more than 20% of the coastal planktonic community and 3–5% in surface ocean waters [7,8] being especially abundant in littoral areas contaminated by hydrocarbons (i.e., marinas and harbours) [9,10]. The analyses of their genomes have revealed the presence in Roseobacters of a great biodegradation potential, mainly for monoaromatic hydrocarbons [11,12]. For example, *Salipiger aestuarii* 357, a member of the Roseobacter lineage isolated from the sands of the coast of Galicia (Northern Spain) that were contaminated due to the accidental oil spill of the Prestige oil tanker in 2002 [13], is capable of growing at the expense of salicylate and naphthalene as sole carbon and energy sources.

## 2. Results

**Figure 2:**
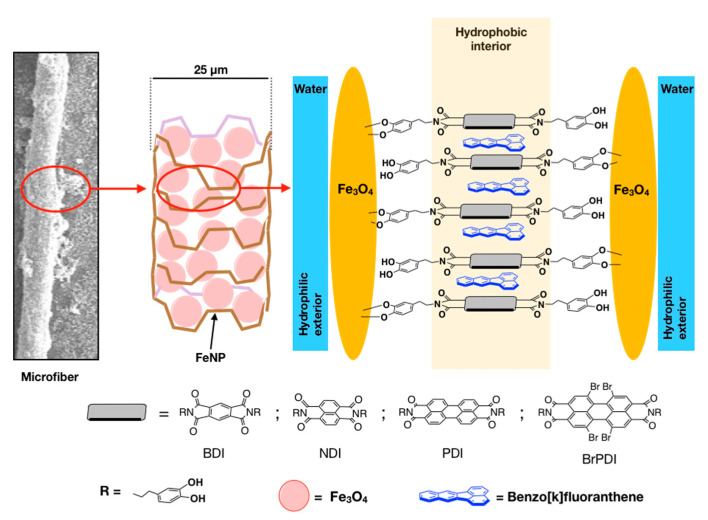
Outline proposed for the formation of microfibres.

The diimide-dopamine ligands were prepared from four electron-deficient π-extended diimides, derived from benzene (BDI), naphthalene (NDI), perylene (PDI), and tetrabromodiimideperylene (BrPDI). The ligands have a symmetrical substitution, with two dopamine units (DA) at both ends, which allow them to be anchored on the surface of the magnetic nanoparticles of Fe_3_O_4_ (FeNP), see Figure 2.

The magnetite nanoparticles (FeNP), used as a nanomaterial, were synthesized by the classical co-precipitation method from Fe (II) and Fe (III) chloride salts [14]. The method of obtaining magnetite is quantitative; it takes place in water, without the use of organic solvents, where magnetite is isolated with the help of a boron-neodymium magnet and without the need to filter or centrifuge, obtaining sodium chloride as a reaction byproduct, an environmentally tolerable contaminant. Magnetite is a natural mineral, which has no storage, handling or disposal problems [15].

The binding between the ligand and the magnetite nanoparticles [16] took place in a basic aqueous solution, using a microwave-ready 5 mL tube. Once sealed, it was irradiated for 30 min between 120–130 °C. Under these reaction conditions, a pressure of 3 bar was reached inside the reaction tube. Increasing the pressure within the reaction caused a greater coating of the Fe_3_O_4_ nanoparticles, substantially increasing the surface coating density and the chemical resistance to extreme aqueous pH conditions. On the contrary, the two hydroxyl groups of the dopamine catechol residues, located in the outermost part of the nanoparticle, formed a hydrophilic layer capable of establishing hydrogen bonds with water and allowed excellent dispersion in these aqueous conditions. These facts were experimentally confirmed by the obtained Dynamic Light Scattering, Zeta Potential Distribution, and Thermogravimetric Analysis (TGA) values (Supplementary Table S1; Supplementary Figures S1–S4).

All nanoreceptors present a polydispersity index (PdI) <0.35 and a low aggregation index. The tests were performed in distilled H_2_O at a temperature of 25 °C and pH = 7.0.

The Langmuir constant was measured to quantify the nature of the association between the PAHs and the nanoreceptor surface. Since all PAHs are fluorescent, the Langmuir constant was determined by fluorescence experiments (Table S2).

The following PAHs (together with their excitation wavelengths) were used to determine de Langmuir constant: naphthalene (λ_exc_ = 270 nm), pyrene (λ_exc_ = 338 nm), benzo[a]pyrene, BAP (λ_exc_ = 266 nm), benzo-k-fluoranthene, BKF (λ_exc_ = 308 nm), dibenzo[ah]anthracene, DB[ah]A (λ_exc_ = 290 nm), and chrysene (λ_exc_ = 285 nm). All tests were carried out in an ethanol:water (1:1) *v*/*v* mixture.

The results obtained for the Langmuir constant between the functionalised nanomaterial and several PAHs of different geometry were excellent, far superior to those described in the literature [17] and similar to those found when using the FeNP-PDI-DA nanomaterial with the same PAHs [18,19] and the same experimental conditions.

To understand the chemical behaviour of PDI and BrPDI, the electrostatic potential (ESP) surfaces of both diimides were computed at the DFT level (B3LYP using 6-31G*) with Spartan,(Wavefunction, Inc., Irvine, California) (see Figure S9). From the inspection of the Figure S9, more positive ESP values (deeper blue zones) were observed for FeNP-BrPDI-DA, located especially between the six central rings of the tetrabromodiimide perylene, suggesting that it was more electron-deficient than FeNP-PDI-DA.

The formation and growth of FeNP-Diimide-DA microfibres occurred spontaneously—thermodynamic process—by adding an aqueous solution of concentration between 10^−5^ M and 10^−6^ M of FeNP-Diimide-DA, within a 6.5–7.0 pH range, to a specific PAH. Due to the slow growth of the fibres, the dispersion was left in a tightly closed vial at room temperature, at rest, and protected from light. After 7 days, the appearance of elongated microscopic fibres was observed, initially attached to the walls of the vial, which eventually grow and disperse within the bulk of the solution. These microfibres were quickly attracted when a neodymium magnet approached the outer wall of the vial (see the video in the Supplementary Material). If a hydrophilic co-solvent was added to the aqueous solution, such as 50% methanol or ethanol (*v*:*v*), the formation of the FeNP-Diimide-DA was slower, and the fibres became visible after 2–3 weeks. Conversely, at concentrations greater than 10^−5^ M, fibre formation was not observed. It was thoroughly verified that, under the same experimental conditions, self-aggregation processes of either diimides or non-functionalised FeNP were not observed.

Given the strength of these aggregates, an energetic acid treatment, such as digestion, was necessary to dissolve and break the fibres into their starting components. This was achieved by treating the fibres with concentrated HCl (37%) at 45 °C for 30 min. From this aqueous-acid residue, the organic phase was extracted with n-heptane [20], and the organic phase was concentrated with water using a dry Ar stream. Subsequently, the extract was solubilised in heptane for its HRMS analysis.

Figures S5–S8 show the high-resolution mass spectra together with the isotopic pattern of PAHs isolated from the digestion of chrysene@FeNP-NDI-DA, BAP@FeNP-NDI-DA, BKF@FeNP-PDI-DA, and DB[ah]A@FeNP-PDI-DA fibres, respectively, which experimentally confirmed the presence of PAHs in the supramolecular complex together with the FeNP-diimide-DA hybrid material.

Fibre formation was dependent on the size of PAH—particularly on the number of double bonds of each PAH molecule [18]—and on the geometry and electronic properties of electron-deficient diimides anchored on the magnetite nanoparticle.

In this study, tubular fibres were observed in the solutions prepared with the dopamine-diimide derivative of benzene FeNP-BDI-DA, with chrysene (nine double bonds), benzo[a]pyrene, BAP (ten double bonds), benzo[k]fluoroanthene, BKF (ten double bonds), and dibenzo[ah]anthracene DB[ah]A (11 double bonds).

**Figure 3:**
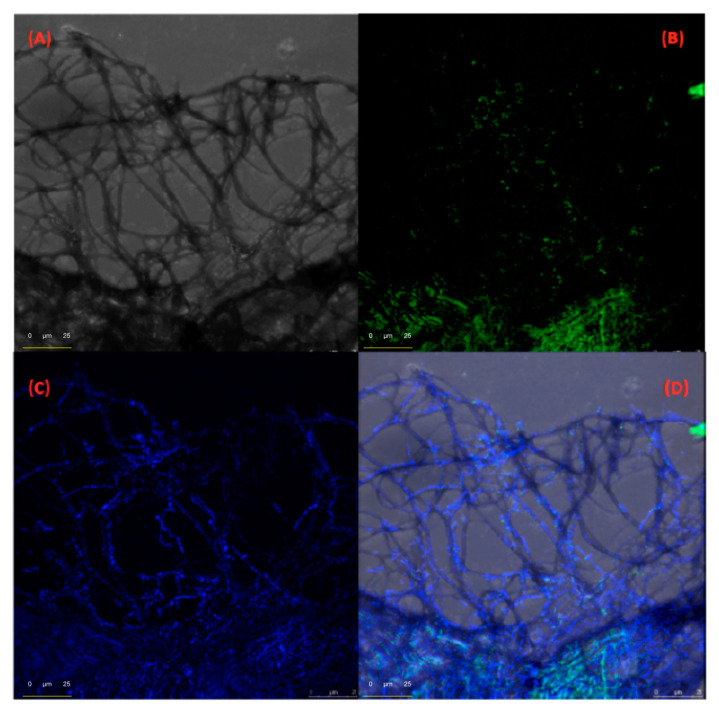
Optical and confocal microphotographs showing microfibres assembled between FeNP-BDI-DA (green) and DB[ah]A (blue) in aqueous media: (**A**) Transmitted light micrograph. (**B**) Fluorescent micrograph λexc = 448 nm, λem = 517 nm (FeNP-BDI-DA). (**C**) Fluorescent micrograph λexc = 405 nm, λem = 454 nm (DB[ah]A). (**D**) Micrograph Composition of A, B, C.

Figure 3 shows a composition of photomicrographs of DB[ah]A@FeNP-BDI-DA network-growing filamentous fibres, performed with the optical microscope (Figure 3A), and with the confocal fluorescent microscope, with a rhodamine laser at λ_exc_ = 540 nm (Figure 3B–D). The distribution of the host and guest in the network is highlighted by the different fluorescent colouration when using different wavelengths for the excitation of the sample. Figure 3B,C shows, preferably in green and blue, the distribution of the FeNP-BDI-DA nano receptor in the network and the distribution of DB[ah]A, respectively. Finally, Figure 3D is the result of superimposing of all photomicrographs.

Likewise, the formation of tubular fibres was observed with the nanoparticles derived from naphthalene diimide, (FeNP-NDI-DA) with pyrene, chrysene, BAP, BKF, and DB[ah]A.

**Figure 4:**
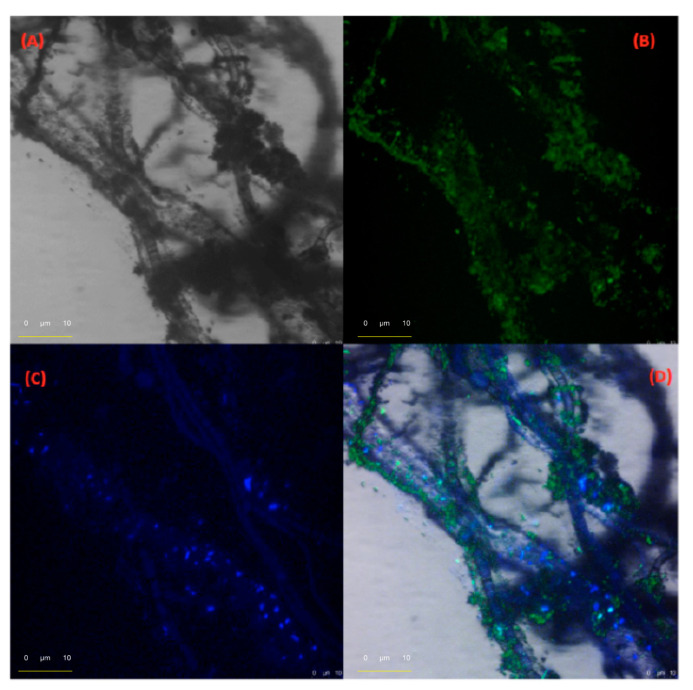
Optical and confocal microphotographs showing microfibres assembled between FeNP-NDI-DA (green) and DB[ah]A (blue) in aqueous media.: (**A**) Transmitted light micrograph. (**B**) Fluorescent micrograph λexc = 448 nm, λem = 517 nm (FeNP-BDI-DA). (**C**) Fluorescent micrograph λexc = 405 nm, λem = 454 nm (DB[ah]A). (**D**) Micrograph Composition of A, B, C.

Figure 4 shows the photomicrographs of the supramolecular assembly formed by the FeNP-NDI-DA with the DB[ah]A, obtained with optical and confocal microscopy. With the hybrid material derived from the tetrabromo perylene diimide, FeNP-BrPDI-DA, flat fibres were observed with chrysene, BKF, BAP, and DB[ah]A.

**Figure 5:**
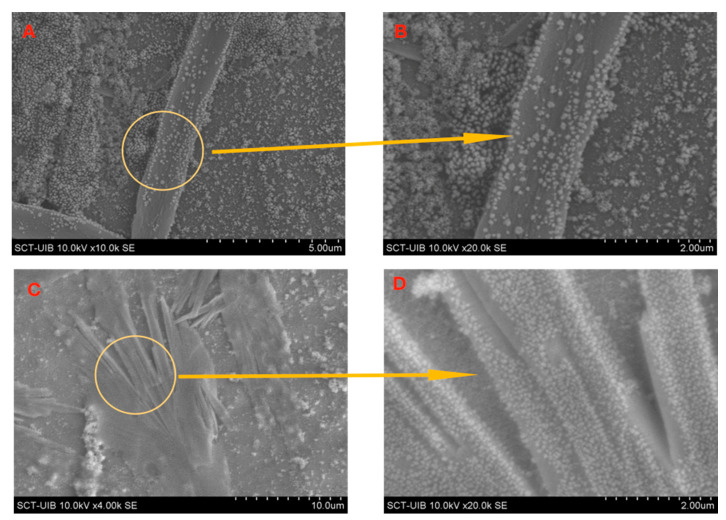
(**A**,**B**) SEM micrographs showing microfibres assembled between FeNP-BrPDI-DA and BKF. (**C**,**D**) FeNP-BrPDI-DA and BHA, in aqueous media.

Figure 5 shows the photomicrographs obtained by SEM for the supramolecular assembly formed by FeNP-BrPDI-DA with BKF and BHA, respectively. From the enlargements of the microphotographs (at 47,000 magnifications), the spherical FeNP nanoparticles of an approximate diameter of 40 nm—light grey—that formed the matrix (skeleton) of the supramolecular complex fibres could be observed.

It is worth noting that, in all cases, the fibres showed great chemical stability in aqueous dispersion, since they remain unchanged over time without showing appreciable alterations up to more than 2 years on the laboratory bench, at room temperature, in a hermetically sealed vial closed and protected from light.

The cooperative degradation method consists, first, of the formation of the naphthalene@FeNP-NDI-DA complex. For this purpose, functionalised magnetic nanoparticles, FeNP-NDI-DA, were added to an aqueous medium contaminated with naphthalene. After 30 min with magnetic stirring, the naphthalene@FeNP-NDI-DA complex was formed, which, if desired, could be separated from the solution by magneto filtration. When integrated into the supramolecular aggregate, naphthalene was placed in a molecularly limited space environment. Then, a second stage follows; after seeding with the *S. aestuarii* 357 strain, it was observed that the degradation of the contaminant was accelerated due to greater availability of the substrate. It is worth mentioning that the environmental impact of magnetite nanoparticles was minimal because it is a biocompatible material [21]. The linker dopamine is not a substance considered dangerous, since it is a neurotransmitter widely distributed in nature and easily biodegradable. The remaining organic components, diimide naphthalene, were degraded by the bacteria without altering their integrity, as evidenced by the conducted experiments.

**Figure 6:**
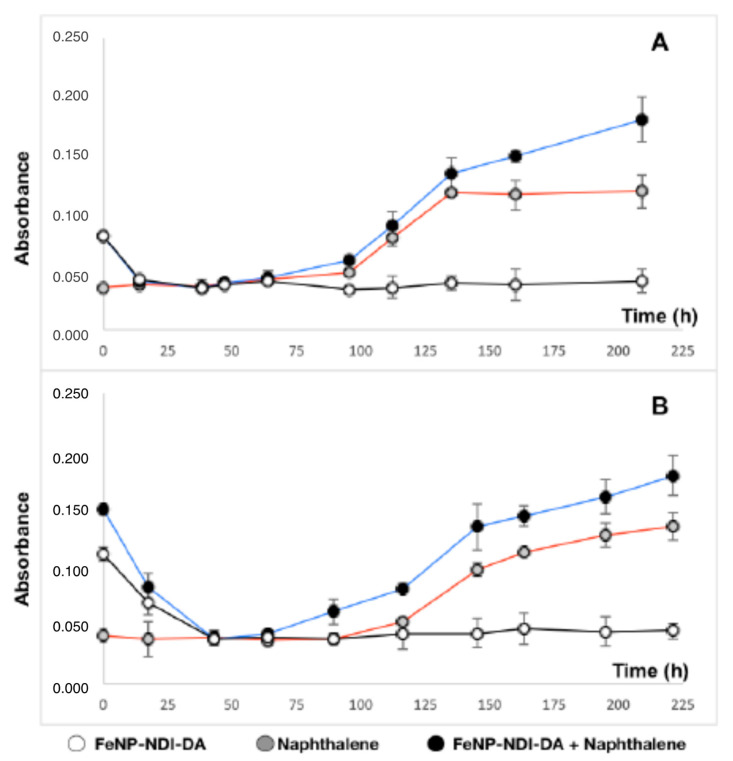
Growth (Absorbance, 595 nm) of *S. aestuarii* 357 with naphthalene (0.1% *w*/*v*) as unique carbon and energy source supplemented with 100 mg/mL (**A**) and 200 mg/mL (**B**) of FeNP-NDI-DA. Growth values are average and SD of three different experimental replicates.

In Figure 6A,B, the growth curves of *S. aestuarii* 357 are shown as a function of time, in three different experimental conditions: in the presence of FeNP-NDI-DA, of naphthalene as the only source of carbon and energy, and of naphthalene and FeNP-NDI-DA (white, grey and black circles, respectively). Figure 6A,B correspond to two different experiments, with different FeNP-NDI-DA nanoreceptor concentrations: 100 mg and 200 mg. The absorbance intensity measurements were recorded at λ_exc_ = 595 nm, and each point represents an average of three experimental replicates.

In these experiments, the bacterium *S. aestuarii* 357 was grown using 100 mL flasks, in 20 mL of a marine mineral medium (see experimental part) [20], fed with naphthalene (0.1%) at 30 °C and under constant agitation (180 rpm). The FeNP-NDI-DA nanoparticles were provided at 1% and 0.5% (*v*/*v*). The growth was monitored by reading absorbances at λ_exc_ = 595 nm.

## 3. Discussion

In these systems, the π-π interactions have most likely taken place between the host and the guest molecules. The strength of the π-π interaction will largely depend on the geometry and electronic properties of both the electron-deficient host (FeNP-Diimide-DA) and the electron-rich guest (PAHs). Therefore, if an electron-poorer host is used, as was the case with the substituted tetrabromo perylene diimide, this interaction will be further favoured. The results obtained for the Langmuir constant support this hypothesis; when comparing the values obtained with the FeNP-BrPDI-DA and its synthetic precursor, the perylene diimide FeNP-PDI-DA, equal or higher (by one order of magnitude) Langmuir constants were observed for the former, in the case of association with pyrene, BKF, DB[ah]A, and chrysene. The computed electrostatic potential also supports this statement.

The biocompatibility of the bacterium *S. aestuarii* 357 with the nanomaterial FeNP-NDI-DA was manifested by being able to grow when naphthalene was added as the sole nutrient, the only source of carbon and energy (Figure 6A,B, grey circles). On the contrary, the bacteria cannot grow if FeNP-NDI-DA (white circles) was the only available compound. The addition of FeNP-NDI-DA nanoparticles (black circles), in the presence of naphthalene, accelerated the growth of *S. aestuarii* 357. This phenomenon was more pronounced the higher the concentration of nanoparticles used (Figure 6A vs. Figure 6B). The non-growth of bacteria with only nanoparticles, and the acceleration of growth with the mixture of nanoparticles and naphthalene, via formation of naphthalene@FeNP-NDI-DA complex, suggests not only that the FeNP-NDI-DA nanoparticles are not toxic to *S. aestuarii* 357—otherwise, either there would be no growth, or it would have been delayed—but, also, they increase the bioavailability of naphthalene, the only source of carbon and energy usable by *S. aestuarii* 357 in our experiments.

## 4. Materials and Methods

### 4.1. General

Reactions were carried out in oven-dried glassware under an atmosphere of argon unless otherwise indicated. Thin-layer chromatography (TLC) was conducted on aluminum plates coated with silica gel (60 F254, Merck, Merck Life Science S.L.U., Madrid, Spain). Column chromatography was performed using silica gel (Geduran Si 60 from Merck (Merck Life Science S.L.U., Madrid, Spain), particle size 0.040–0.063 mm) as a stationary phase.

### 4.2. Materials

All commercially available reagents: dopamine hydrochloride, triethylamine, perylene and 3,4,9,10-tetracarboxylic dianhydride, were supplied by Sigma–Aldrich (Merck Life Science S.L.U., Madrid, Spain). All the solvents were purchased from Scharlab (Scharlab, S. L., Barcelona, Spain).

### 4.3. Instrumentation

^1^H and ^13^C NMR spectra were recorded on a Bruker Advance Spectrometer (Bruker Española S.A., Madrid, Spain) at 300 and 75 MHz at 25 °C. Chemical shifts are reported as a part per million (δ, ppm) referenced to the residual protium signal of deuterated solvents. Spectral features are tabulated in the following order: chemical shift (δ, ppm), multiplicity (s-singlet, d-doublet, t-triplet, and m-multiplet), number of protons. (FTIR) were obtained on Bruker Tensor 27 (Bruker Española, Madrid, Spain) instrument in solid-state. Matrix-assisted laser desorption/ionisation mass spectra (MALDI) were recorded with an Autoflex III MALDI TOF/TOF mass spectrometer provided with a Smartbeam Laser at 200 Hz (Bruker Española, Madrid, Spain). Functionalization of iron nanoparticles was performed on a Biotage Initiator Classic Microwave Synthesizer (Biotage, NASDAQ, Stockholm) at 400 W and 2 bar.

### 4.4. Synthesis of Magnetite Nanoparticles (FeNP)

For the synthesis of Fe_3_O_4_ nanoparticles, an adapted procedure of F. Yazdani et al. [14] was used. At room temperature, 50 mL FeCl_3_ of 0.1 M solution and 25 mL FeCl_2_ of 0.1 M solution were added to 350 mL distilled deionised water under an argon atmosphere and magnetically stirred. Then 35 mL of the 1 M NaOH solution was added to the reaction vessel and the mixture stirred for 3 min. The black precipitated product was separated with a boron–neodymium magnet. The precipitate was washed 3 times with 50 mL deionised water. After washing, the black product was dried in a vacuum oven for 12 h at 80 °C.

FTIR (KBr): 3406, 2921, 2851, 1591, 1384, and 632 cm^−1^.

### 4.5. Preparation of Ligands

Preparation of 1,6,7,12-tetrabromoperylene-3,4,9,10-tetracarboxylic acid bisanhydride [21]: The bromination of perylene-3,4,9,10-tetracarboxylic acid bisanhydride, was carried out with Br_2_ in a mixture of sulfuric acid and fuming sulfuric acid. Then, 0.516 g of perylene-3,4,9,10-tetracarboxylic acid bisanhydride, (1.31 mmol) was dissolved in a mixture of 6 mL of sulfuric acid 98 wt% with 1 mL of fuming sulfuric acid, under magnetic stirring for 12 h. After the 12 h, the temperature was raised to 80 °C, and 7.75 mg of iodine (0.03 mmol) and 150 µL of bromine were added dropwise (2.63 mmol). The mixture was heated for 48 h at 80 °C. Then, 7.75 mg of iodine (0.03 mmol) was added, and the temperature was raised to 100 °C, and 150 µL of bromine was added again dropwise. The mixture was heated at 100 °C for 80 h. Finally, the brown-oil was poured onto ice water, and a dark red precipitate was observed. The solid was filtered and washed with 10 mL of sulfuric acid. The red solid was washed with miliQ water until the filtrate became neutral. The product obtained, as a dark red powder, was dried in a vacuum at 120 °C for 8 h, giving 0.409 g (44%, 0.578 mmol). The crude product was used directly in the next step without further purification. ^1^H NMR (300 MHz, DMSO-*d*_6_). δ: 8.74 (4H, s). FTIR (KBr): ν = 3442, 1716, 1634, 1591, 1557, 1431, 1361, 852, 810, 768 cm^−1^_._ HRMS (EI): m/z = 708 (M^+^).

Preparation of 2,9-bis(3,4-dihydroxyphenethyl)-1,6,7,12-tetrabromoperylene tetracarboxylic bisimide: In a round bottom flask 0.176 g (0.250 mmol) of 1,6,7,12-tetrabromoperylene-3,4,9,10-tetracarboxylic acid bisanhydride was dissolved in 15 mL of a 1:1 (*v*/*v*) mixture of H_2_O and DMF. Then, 2 mL of Et_3_N was added and stirred for 2 h plus. After that, 99 mg (0.522 mmol) of dopamine hydrochloride was dissolved in 10 mL of a 1:1 (*v*/*v*) mixture of H_2_O and DMF and was added dropwise to the first flask. Once the addition was complete, the mixture was refluxed overnight. The oil obtained was allowed to temper and was transferred to a falcon tube, where concentrated HCl was added until a brown–reddish precipitate was formed. The crude was centrifuged and washed repeatedly with miliQ water, by centrifugation, until neutrality of the supernatant liquid was observed. The product was dried in a vacuum at 120 °C for 8 h and obtained as a reddish–brown powder, 98 mg (0.100 mmol, 40% yield). ^1^H NMR (300 MHz, DMSO-d_6_): δ: 8.7 (s, 4H), 7.9 (s, 4H), 6.90 (m, 6H), 3.65 (s, 4H), 2.67 (t, 4H). ^13^C NMR (300 MHz, DMSO-d_6_). δ: 176.9 (C=O), 137.7 (C=C), 132.4 (C-Br), 128.3 (C=C), 127.9 (C=C), 127.7 (C=C), 120.8 (C=C), 45.3 (CH_2_), 41.7 (CH_2_). FTIR (KBr): ν = 3442, 1716, 1634, 1591, 1557, 1431, 1361, 852, 810, 768 cm^−1^_._ MALDI-TOF-MS m/z (%): [M]^+^ calculated. for C_40_H_22_Br_4_N_2_O_8_ 978.2380 found 977.8069.

### 4.6. Preparation of Functionalised Magnetite Nanoparticles

In a microwave tube 25.26 mg (0.026 mmol) of 2,9-bis(3,4-dihydroxyphenethyl)-1,6,7,12-tetrabromoperylene tetracarboxylic bisimide was introduced with 4 mL of MiliQ water, one drop of 1M NaOH and 1 mL of magnetite nanoparticles suspension (11.4 mg/mL). The mixture was sonicated for 5 min and then introduced in the microwave reactor. The reaction conditions of the microwave reactor were: 120 °C, 3 bar and 30 min of reaction time. Once the reaction was over, the nanoparticles were decanted with the help of a boron-neodymium magnet and washed 3 times with EtOH. Finally, the hybrid nanomaterial was suspended and stored in 10 mL of EtOH in an argon atmosphere.

FTIR (KBr): *ν* = 3442 (s), 1692 (s), 1643 (m), 1594 (s), 1517 (s), 1403 (s), 1385 (s), 1319 (s), 672 (s) and 588 cm^−1^ (s).

### 4.7. General Procedure for the Formation of Microfibres

The preparation of magnetic microfibres was carried out as follows: Two hundred microlitres of a 1.14 mg/mL suspension of magnetite nanoparticles functionalised with one of the FeNP-diimide-DA receptors was introduced into a 10 mL vial. Afterward, 4.70 mL of miliQ water was added to the vial. To this solution, 100 µL of a 10^−4^ M solution of chrysene, benzo[a]pyrene, benzo-k-fluoranthene, or dibenzo[a,h]anthracene dissolved in a mixture of water/ethanol, 1:3 (*v*/*v*) was added, respectively. The vial was tightly closed and was left at room temperature, in the dark, for 14 days. Enough time for microfibres to develop and observed with the naked eye. After this time, no further growth occurred. The final concentrations were: functionalised nanoparticles: 0.456 mg/mL, and polycyclic aromatic hydrocarbon, PAHs: 10^−6^ M.

### 4.8. Growth Curves of S. aestuarii 357

The bacterium *S. aestuarii* 357 was grown using 100 mL flasks, in 20 mL of a marine mineral medium (see experimental part) [20], fed with naphthalene (0.1%) at 30 °C, under constant agitation (180 rpm). The FeNP-NDI-DA nanoparticles were provided at 1% and 0.5% (*v*/*v*). The growth was monitored by reading absorbances at λ_exc_ = 595 nm.

## 5. Conclusions

This study showed that in the global assembly process of magnetic nanoparticles modified with PAHs, resistant supramolecular aggregates are always formed, which in some cases can form fibres of up to several hundreds of micrometres in length, most likely due to the π-π interactions established between PAHs and the electron-deficient surface of the diimides anchored to FeNP, as can be inferred from ESP calculations. On the other hand, it was shown that the formation of the supramolecular aggregates with naphthalene, the most abundant PAH pollutant in the environment, promotes bioavailability, and accelerates the degradation of naphthalene. These results allow us to propose, as a strategy for the removal and biodegradation of PAHs from contaminated aqueous media, the combined use of FeNP-diimides-DA together with a marine microorganism, such as *S. aestuarii* 357, belonging to the bacterial lineage of Roseobacter, as a sustainable measure of environmental decontamination, based on the capture, sequestration, and biodegradation of toxic pollutants.

## Data Availability

Not applicable

## Supplementary Materials

Supplementary materials can be found at https://www.mdpi.com/1422-0067/22/1/17/s1.
