# Supplementary material for: Effective Elimination and Biodegradation of Polycyclic Aromatic Hydrocarbons from Seawater through the Formation of Magnetic Microfibres
Source: Int J Mol Sci. 2020 Dec 22;22(1):17. doi: 10.3390/ijms22010017 (PMC7792786; doi:10.3390/ijms22010017)
Supplement: Supplementary file 1 [file ijms-22-00017-s001.pdf]

Supplementary material

Effective elimination and biodegradation of polycyclic aromatic hydrocarbons from seawater through the formation of magnetic microfibers.

M. Susana Gutiérrez,[1] Alberto J. León,[1] Paulino Duel,[1] Rafael Bosch,[2,3]* M. Nieves Piña[1,]* and Jeroni Morey[1]

[1] Department of Chemistry, University of the Balearic Islands, Crta. de Valldemossa, Km. 7.5, 07122 Palma de Mallorca, Balearic Islands, Spain.
[2] Department of Biology, University of the Balearic Islands, Crta. de Valldemossa, Km. 7.5, 07122 Palma de Mallorca, Balearic Islands, Spain.
[3] Environmental Microbiology, IMEDEA (CSIC-UIB), Miquel Marquès, 21, 07190 Esporles, Balearic Islands, Spain.
* Correspondence: neus.pinya@uib.es; Tel.: +34-971-172847

1. Dynamic Light Scattering (DLS). Zeta potential Values and Thermogravimetric (TGA) of Fe_3O_4 nanoparticles diimides-dopamine functionalized.

Hybrid nanomaterial	DLS (nm)	Zeta Potential (mV)	Weight loss (TGA, %)
**FeNP-BDI-DA**	295.0	-43.3	45.15
**FeNP-NDI-DA**	300.6	-38.3	44.07
**FeNP-PDI-DA**	335.2	-51.3	79.90
**FeNP-BrPDI-DA**	225.9	-42.6	61.44

Table S1. Dynamic Light Scattering (DLS), Zeta potential Values and Thermogravimetric (TGA) of Fe_3O_4 nanoparticles diimides-dopamine functionalized.

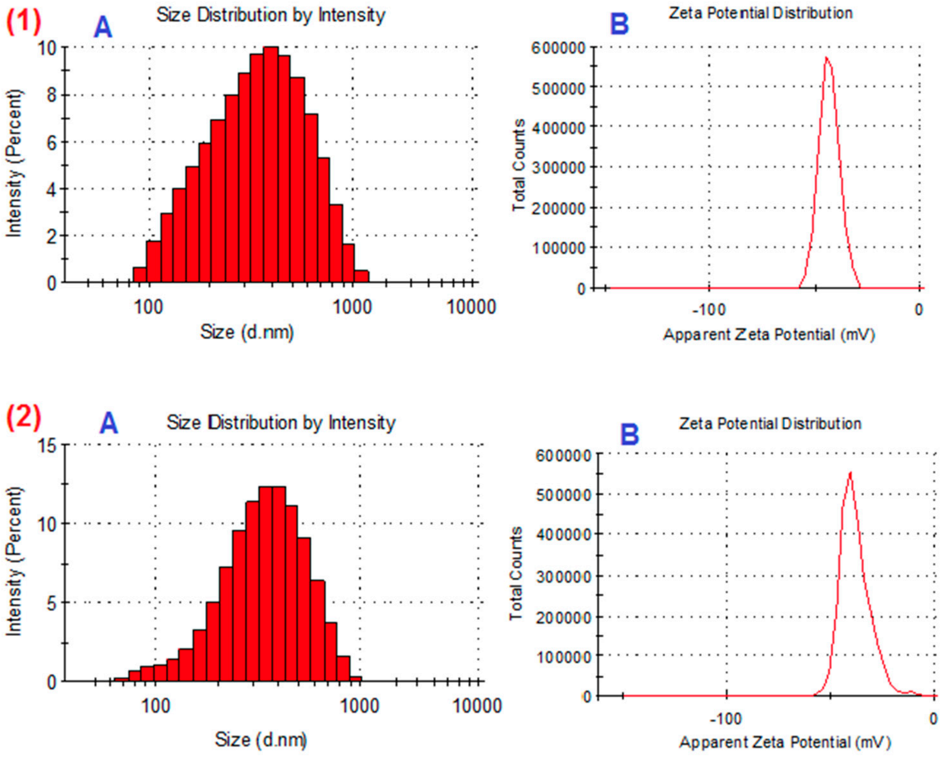

Figure S1. DLS size distribution (A) and Zeta potential (B) of (1) FeNP-BDI-DA, and (2) FeNP-NDI-DA in H_2O (pH=7.0) at 25°C.

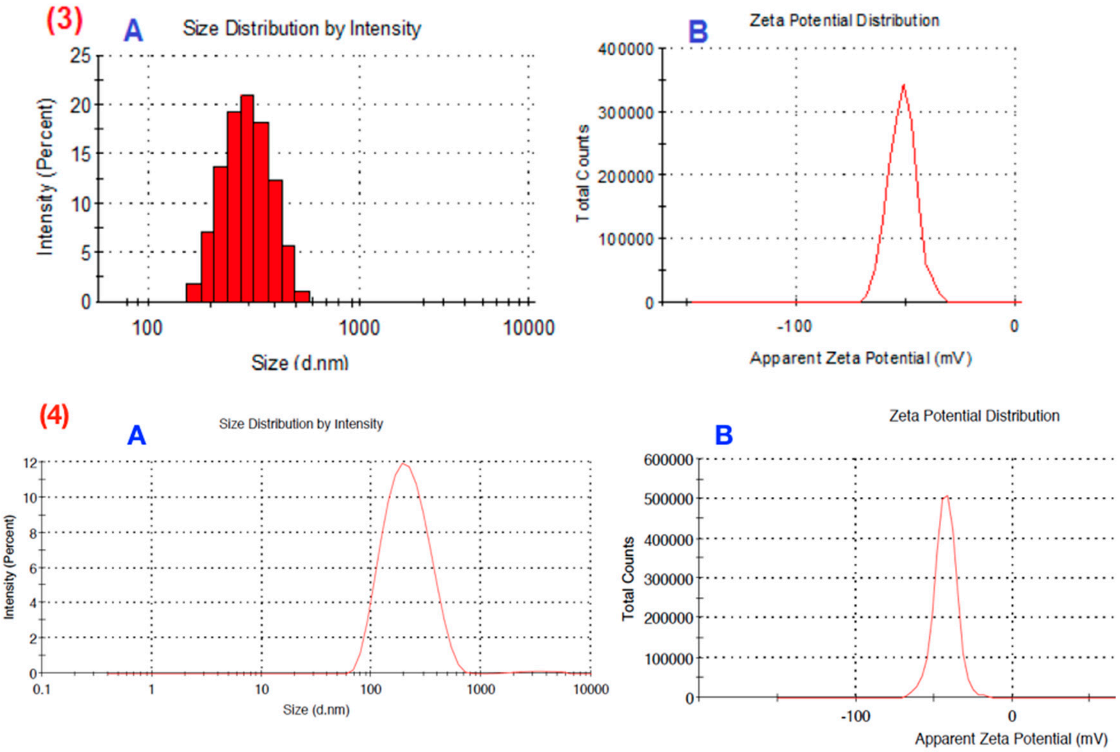

Figure S2. DLS size distribution (A) and Zeta potential (B) of (3) FeNP-PDI-DA, and (4) FeNP-BrPI-DA in H_2O (pH=7.0) at 25 °C.

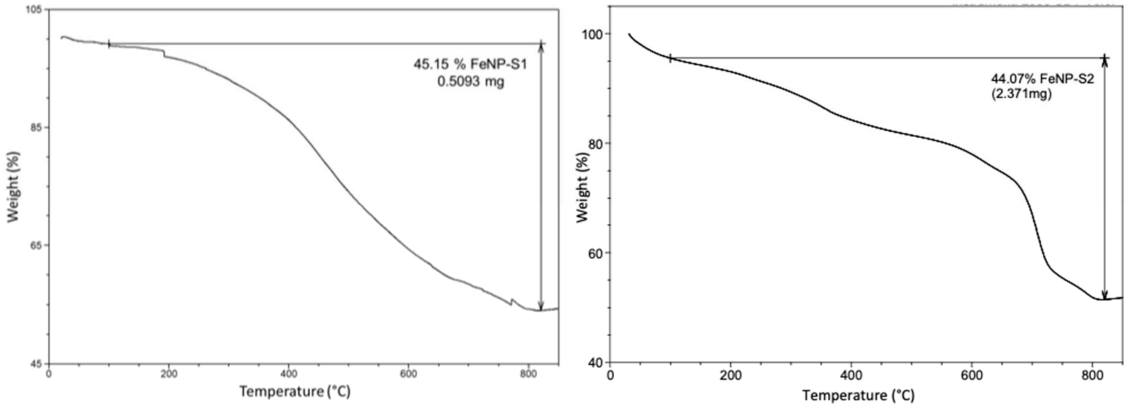

Figure S3. Thermogravimetric analysis (TGA) for FeNP-BDI-DA and FeNP-NDI-DA.

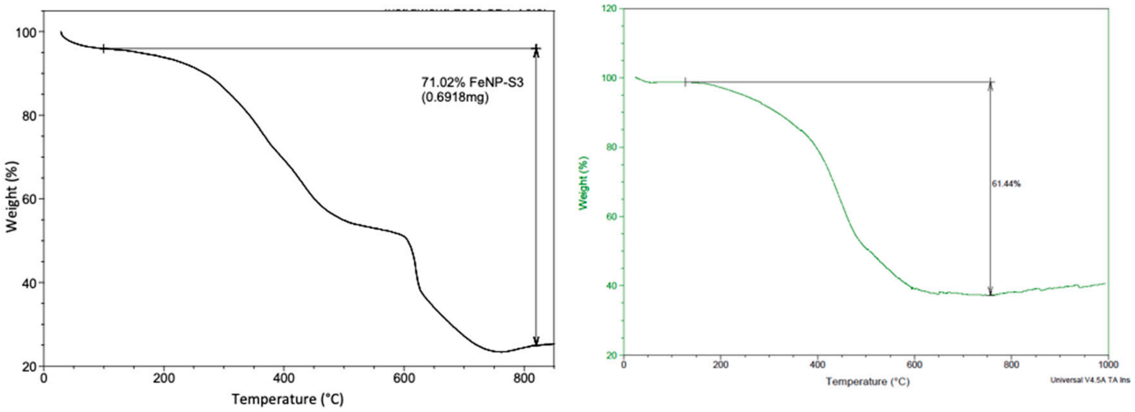

Figure S4. Thermogravimetric analysis (TGA) for FeNP-PDI-DA and FeNP-BrPDI-DA.

2. Determination of Langmuir Constant

Four solutions of known concentration of PAHs (4 x 10^{-7} M, 6 x 10^{-7} M, 8 x 10^{-7} M and 1 x 10^{-6} M) in 1: 1 v / v EtOH / H_2O, are maintaining in contact with four different amounts of FeNP-Diimide-DA, - 0.01 mg, 0.02 mg, 0.03 mg, 0.04 mg – for the saturation is occurred. The solutions are stored for 4 hours, with sporadic agitation, in the dark. Then they are separated with a neodymium magnet ($Nd_2Fe_{14}B$, exhibiting 11 kOe of magnetic field), and the fluorescence intensity is determined, calculating the final concentration of the solution using a calibration curve, performed under the same conditions and simultaneously. The slope of each line, obtained from the Langmuir representation with each quantity of solid, indicates the degree of interaction between the FeNP-Diimide-DA solid and the PAHs used. To obtain a statistically significant mean of the Langmuir equilibrium constant the experiment is repeated at least 3 times.

Hybrid Nanomaterial + PAHs	Langmuir Constant (K), M^{-1}
**FeNP-BDI-DA + Naphthalene**	(3.9 ± 0.2) x 10^5
**FeNP-NDI-DA + Naphthalene**	(4.8 ± 0.3) x 10^6
**FeNP-BrPDI-DA + Naphthalene**	(1.9 ± 0.1) x 10^6
**FeNP-PDI-DA + Naphthalene**	(4.5 ± 0.5) x 10^6
**FeNP-BDI-DA + Pyrene**	(6.0 ± 1.1) x 10^5
**FeNP-NDI-DA + Pyrene**	(2.4 ± 0.4) x 10^6
**FeNP-BrPDI-DA + Pyrene**	(1.0 ± 0.2) x 10^6
**FeNP-PDI-DA + Pyrene**	(2.0 ± 0.3) x 10^5
**FeNP-BDI-DA + DB[ah]A**	(6.6 ± 0.9) x 10^5
**FeNP-NDI-DA + DB[ah]A**	(7.6 ± 1.1)x 10^6
**FeNP-BrPDI-DA + DB[ah]A**	(1.3 ± 0.1) x 10^6
**FeNP-PDI-DA + DB[ah]A**	(4.8 ± 0.5) x 10^5
**FeNP-BrPDI-DA + BKF**	(1.1 ± 0.2) x 10^6
**FeNP-PDI-DA + BKF**	(4.4 ± 0.3) x 10^5
**FeNP-BrPDI-DA + Chrysene**	(2.3 ± 0.2) x 10^6
**FeNP-PDI-DA + Chrysene**	(3.5 ± 0.3) x 10^5

Table S2. Langmuir constants calculated for the different hybrid nanomaterial and PAHs tested.

3. HRMS Supramolecular Complexes

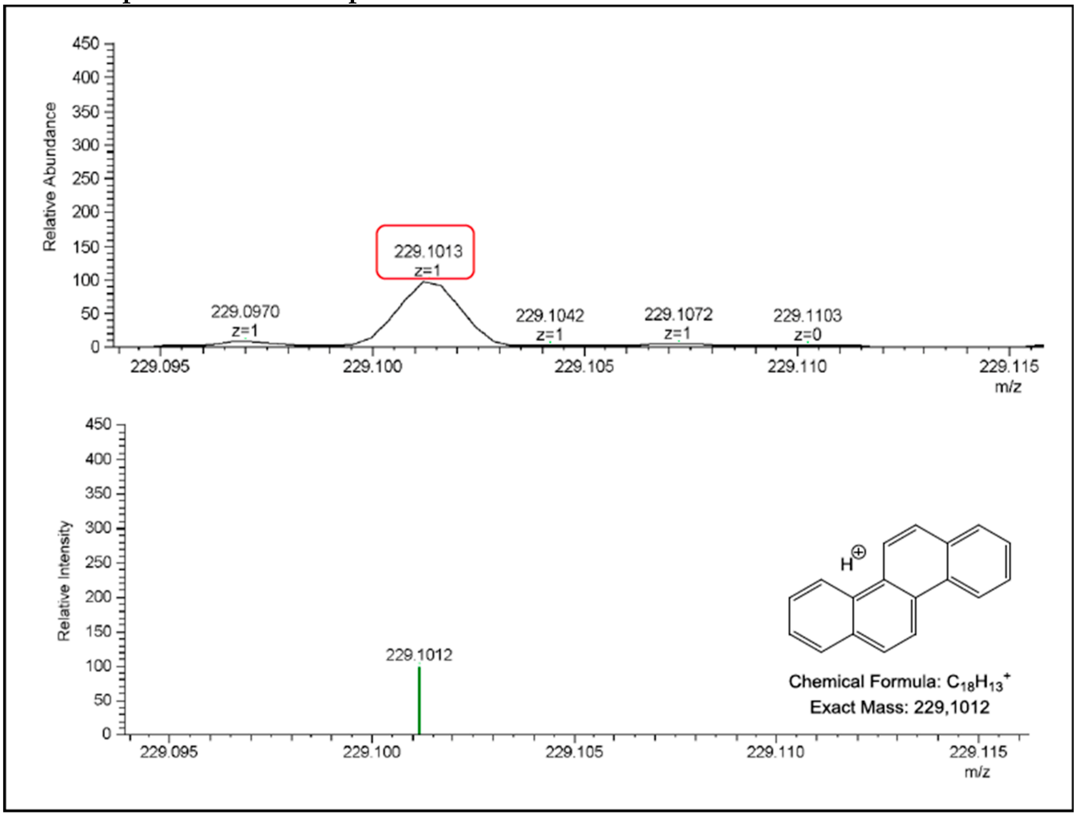

Figure S5. HRMS analysis of the microfiber digestion of FeNP-NDI-DA with Chrysene.

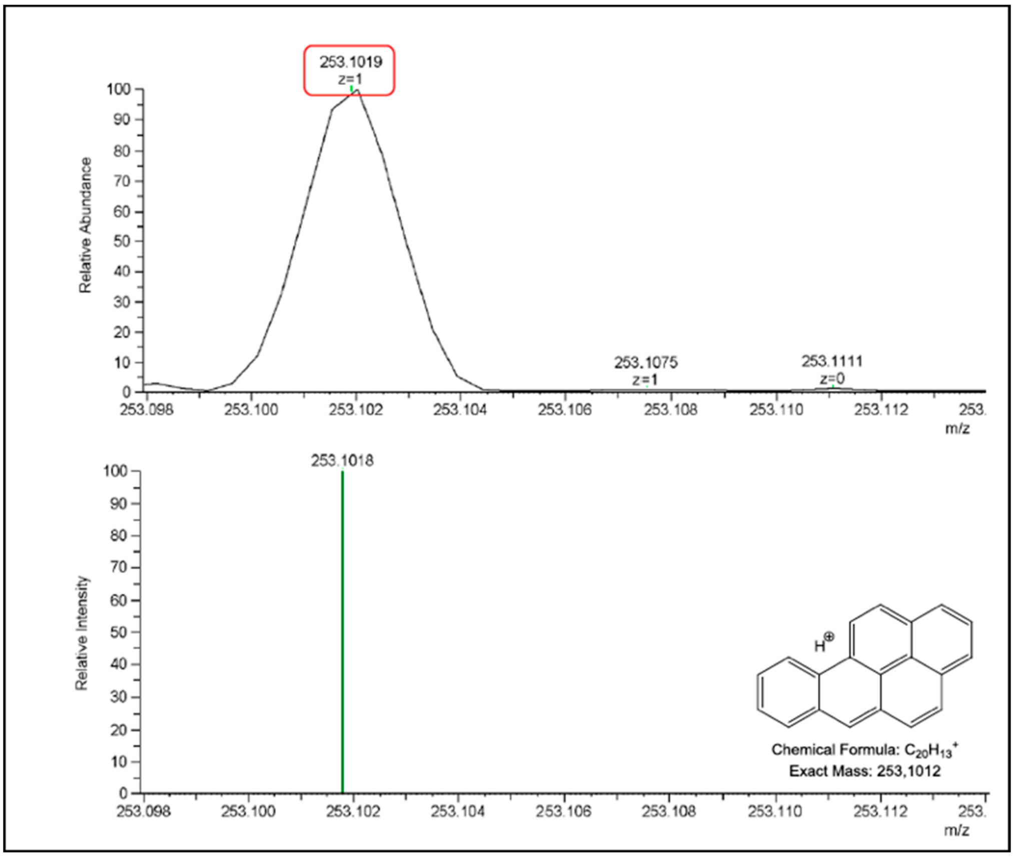

Figure S6. HRMS analysis of the microfiber digestion of FeNP-NDI-DA with BAP.

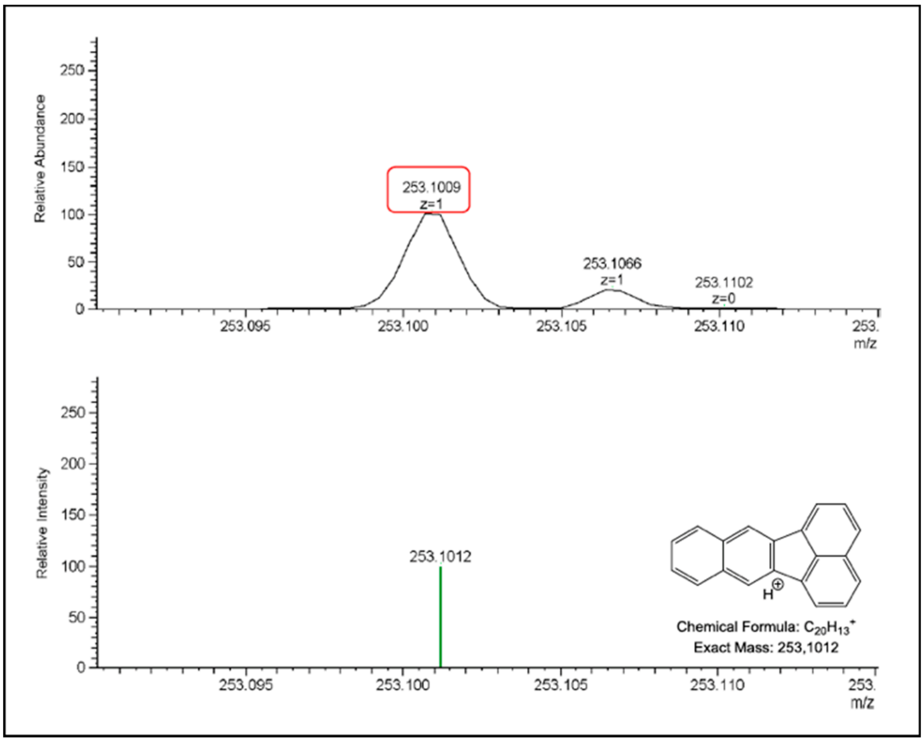

Figure S7. HRMS analysis of the microfiber digestion of FeNP-PDI-DA with BKF.

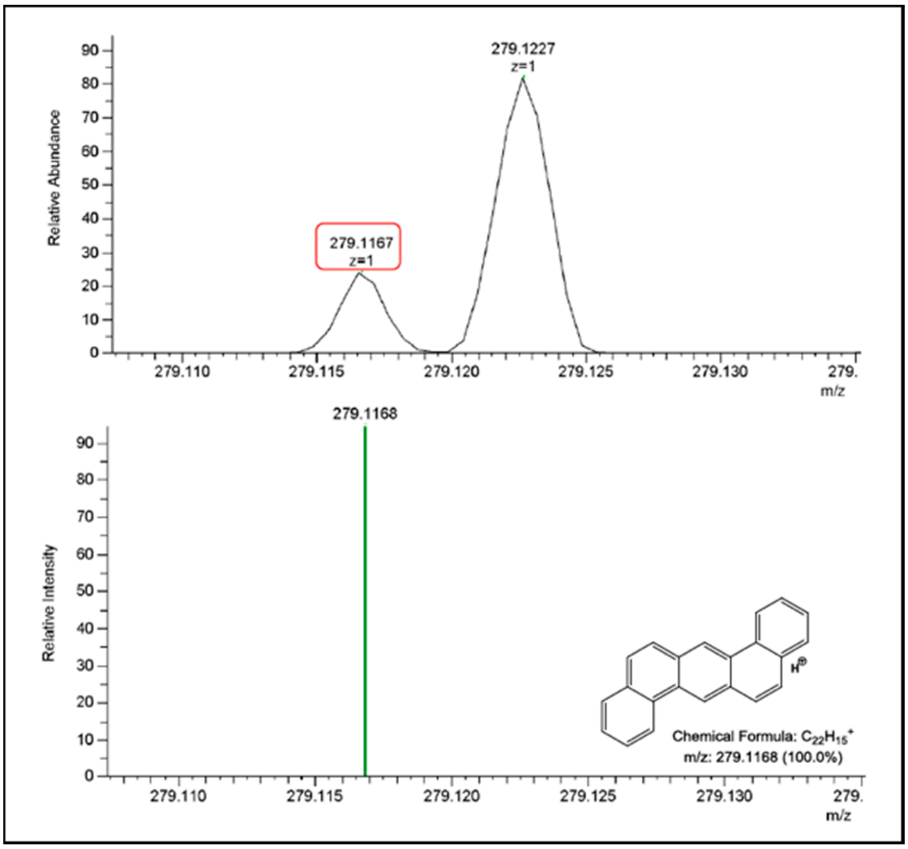

Figure S8. HRMS analysis of FeNP-PDI-DA microfiber digestion with DB[ah]A.

4. Biodegradability and Biocompatibility Experiments

The composition similar seawater consists of the following salts:
Na_2HPO_4 (1.785 g/l). KH_2PO_4 (0.750 g/l). NH_4Cl (0.500 g/l) $MgSO_4.7H_2O$ (0.100 g/l). Citrate Ferric Ammonium (0.250 mg/l). $CaCl_2$ (0.375 mg/l). $ZnSO_4.7H_2O$ (0.050 mg/l). $MnCl_2.4H_2O$ (0.015 mg/l). H_3BO_3 (0.150 mg/l)
$CoCl_2.6H2O$ (0.100 mg/l). $CuCl_2.2H_2O$ (0.005 mg/l). $NiCl_2.6H_2O$ (0.010 mg/l).
$Na_2MoO_4.2H_2O$ (0.015 mg/l).

5. Electrostatic Potential Surfaces

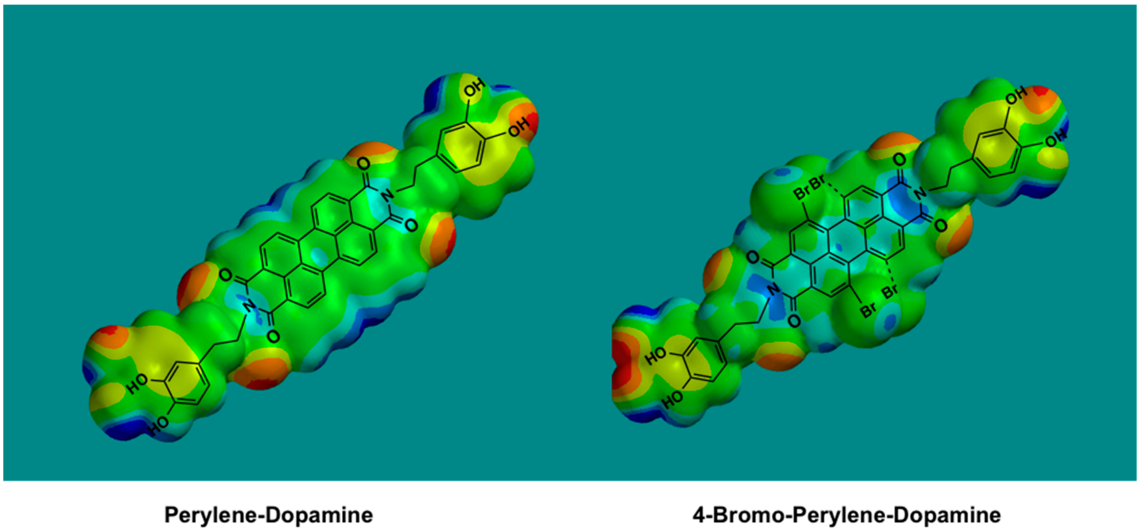

Figure S9. The electrostatic potential surfaces for the two ligands, were plotted from DFT calculations (B3LYP using 6-31G*) with Spartan, (Wavefunction, Inc.).
